# Personal

**Published:** 1895-06

**Authors:** 


### Personal.

Dr. Allen McLane Hamilton, of New York, sailed for England Saturday, May 11, 1895. It is announced that it is Dr. Hamilton's intention to live in London hereafter during May, June, July and August, the London season. He has rented a house for that period and will open an office. A considerable part of Dr. Hamilton's practice has for a long time been among foreigners and Americans that live abroad, and his opening an office in London has been in response to oft-repeated requests on the part of those clients. He will practise in New York the other part of the year, arriving home when his patients begin to return from the summer outings, so that his home practice will not be interfered with. The precedent of American physicians practising part of the year in London was established, we believe, by Dr. S. Weir Mitchell.

Dr. Henry J. Mulford, of Buffalo, has removed from 56 Allen street to 466 Franklin street, where he will continue to give special attention to diseases of the nose and throat. Hours 9 to 12.

Dr. George W. Pattison, of Buffalo, has removed from 17 Court street to 22 West Chippewa street.

Dr. Frank J. Thornbury was elected president of the Buffalo Microscopical Society at the annual meeting held April 8, 1895.

